# Secondary Metabolites in the *Dendrobium heterocarpum* Methanolic Extract and Their Impacts on Viability and Lipid Storage of 3T3-L1 Pre-Adipocytes

**DOI:** 10.3390/nu14142886

**Published:** 2022-07-14

**Authors:** Sakan Warinhomhoun, Hnin Ei Ei Khine, Boonchoo Sritularak, Kittisak Likhitwitayawuid, Tomofumi Miyamoto, Chiaki Tanaka, Chuchard Punsawad, Yanyong Punpreuk, Rungroch Sungthong, Chatchai Chaotham

**Affiliations:** 1 School of Medicine, Walailak University, Nakhon Si Thammarat 80160, Thailand; sakan.cu@gmail.com (S.W.); chuchard.pu@wu.ac.th (C.P.); 2 Center of Excellence in Marijuana, Hemp, and Kratom, Walailak University, Nakhon Si Thammarat 80160, Thailand; 3 Department of Pharmacognosy and Pharmaceutical Botany, Faculty of Pharmaceutical Sciences, Chulalongkorn University, Bangkok 10330, Thailand; boonchoo.sr@chula.ac.th (B.S.); kittisak.l@chula.ac.th (K.L.); 4 Department of Biochemistry and Microbiology, Faculty of Pharmaceutical Sciences, Chulalongkorn University, Bangkok 10330, Thailand; hnineieikhine12@gmail.com (H.E.E.K.); rungroch.s@chula.ac.th (R.S.); 5 Natural Products for Ageing and Chronic Diseases Research Unit, Faculty of Pharmaceutical Sciences, Chulalongkorn University, Bangkok 10330, Thailand; 6 Graduate School of Pharmaceutical Sciences, Kyushu University, Fukuoka 812-8582, Japan; miyamoto@phar.kyushu-u.ac.jp (T.M.); ctanaka@wakayama-med.ac.jp (C.T.); 7 School of Pharmaceutical Sciences, Wakayama Medical University, Wakayama 640-8156, Japan; 8 Department of Agriculture, Kasetsart University, Bangkok 10900, Thailand; cyyp01@hotmail.co.th; 9 Preclinical Toxicity and Efficacy Assessment of Medicines and Chemicals Research Unit, Faculty of Pharmaceutical Sciences, Chulalongkorn University, Bangkok 10330, Thailand

**Keywords:** *Dendrobium heterocarpum*, phytochemical, bibenzyl, adipocyte differentiation, obesity

## Abstract

Although many natural products have proven their potential to regulate obesity through the modulation of adipocyte biology, none of them has yet been approved for clinical use in obesity therapy. This work aims to isolate valuable secondary metabolites from an orchid species (*Dendrobium heterocarpum*) and evaluate their possible roles in the growth and differentiation of 3T3-L1 pre-adipocytes. Six compounds were isolated from the orchid’s methanolic extracts and identified as amoenylin (**1**), methyl 3-(4-hydroxyphenyl) propionate (**2**), 3,4-dihydroxy-5,4’-dimethoxybibenzyl (**3**), dendrocandin B (**4**), dendrofalconerol A (**5**), and syringaresinol (**6**). Among these phytochemicals, compounds **2**, **3**, and **6** exhibited lower effects on the viability of 3T3-L1 cells, offering non-cytotoxic concentrations of ≲ 10 µM. Compared to others tested, compound **3** was responsible for the maximum reduction of lipid storage in 3T3-L1 adipocytes (IC_50_ = 6.30 ± 0.10 µM). A set of protein expression studies unveiled that compound **3** at non-cytotoxic doses could suppress the expression of some key transcription factors in adipocyte differentiation (i.e., PPARγ and C/EBPα). Furthermore, this compound could deactivate some proteins involved in the MAPK pathways (i.e., JNK, ERK, and p38). Our findings prove that *D*. *heterocarpum* is a promising source to explore bioactive molecules capable of modulating adipocytic growth and development, which can potentially be assessed and innovated further as pharmaceutical products to defeat obesity.

## 1. Introduction

As a critical risk factor that correlates to various pathological conditions and chronic diseases, obesity turns out to be a public health concern worldwide. In 2020, the World Health Organization reported that 39% and 13% of adults (age > 18 years) were overweight and obese, respectively [1]. Irregular adipose tissue formation due to excess growth and development of adipocytic cells is a sign of obesity. This uneven biological process does not induce obesity only but also consequently provokes other diseases such as cancers, diabetes mellitus, cardiovascular diseases, chronic kidney diseases, and metabolic syndrome [2,3,4]. Therefore, modulating adipocytic growth and development by pharmaceutical products has become a considerable research hotspot for regulating obesity in these recent years [5].

Adipogenesis is a complex multistep process to convert pluripotent stem cells to mature adipocytes [6]. One of the critical steps in adipogenesis is the cellular lipogenesis triggered by some transcription factors (e.g., peroxisome proliferators activated receptor γ (PPARγ) and CCAAT/enhancer-binding protein α (C/EBPα)) during adipocyte differentiation [7]. Repressing the expression of these transcription factors can disrupt adipocyte differentiation and cellular lipid accumulation [8]. The other molecular events that occur instinctively in cells (including adipocytic cells) are the mitogen-activated protein kinase (MAPK) signaling pathways that involve several kinase cascades, mainly c-Jun N-terminal kinase (JNK), extracellular signal-regulated kinase (ERK), and stress-activated protein kinase (p38) [9,10]. Deactivation of these upstream regulators has been proposed to have some potential links to the suppressed adipocyte differentiation [11], which offers a promising molecular mechanism to modulate obesity.

Although some available anti-obesity drugs have been revealed for their success in weight loss, they still pose various adverse effects, such as cardiovascular toxicity, hallucinations, headache, anxiety, and hepatic toxicity [11]. In recent years, natural compounds with a high safety profile, particularly those derived from plants, have gained more attention due to their inhibitory activity in adipocyte differentiation and obesity development [12,13]. However, none of the discovered natural products have been approved for clinical use in obesity therapy. Moreover, only a few percentages of these compounds have been unveiled for their suppressive function in adipocyte differentiation and anti-obesity potential.

*Dendrobium* is one of the largest genera in the family *Orchidaceae*, consisting of approximately 1400 member species. More than 1100 species are predominant in Asia and Australia, of which approximately 150 species are omnipresent in Thailand [14,15]. *Dendrobium* plants are great sources for anti-diabetes mellitus [16,17,18,19,20,21,22,23,24,25,26], but their roles in the differentiation and cellular response of adipocytic cells are still scarce [20,26,27]. *Dendrobium heterocarpum* Wall. ex. Lindl, so-called “Ueang Si Tan (in Thai)”, is another prevalent *Dendrobium* species in Thailand [28], while its phytoconstituents and pharmaceutical properties are still waiting to explore.

In this study, we aimed to profile secondary metabolites in the methanolic extracts derived from the whole *D. heterocarpum* plants and assess how the identified compounds exert their roles in the viability and differentiation of the mouse embryonic 3T3-L1 pre-adipocytes. The biological impacts of such phytochemicals on cellular lipid storage and some underlying molecular mechanisms potentially linked to obesity incidence and therapy were elucidated and discussed.

## 2. Materials and Methods

### 2.1. Plant Materials

The whole *D*. *heterocarpum* plant was purchased from Chatuchak market in Bangkok, Thailand (June 2019). The plant specimen was identified and confirmed by a curator, Mr. Yanyong Punpreuk, using the database available at the Botanical Garden Organization. A specimen voucher (BS-Dhet-012562) was generated and deposited at the department of Pharmacognosy and Pharmaceutical Botany, Faculty of Pharmaceutical Sciences, Chulalongkorn University, Thailand.

### 2.2. Extraction, Fractionation, and Phytochemical Elucidation

The dried plant material (3.6 kg) was extracted with MeOH (4 × 15 L), and the methanolic extract (300.6 g) was partitioned in a mixture of H_2_O, EtOAc, and *n*-butanol to give aqueous (105.24 g), ethyl acetate (126.59 g), and *n*-butanol (112.78 g) extracts. The ethyl acetate extract was partitioned in hexane-20% H_2_O/MeOH to obtain hexane (11.0 g) and methanolic (16.5 g) extracts. The methanolic extract was subsequently fractioned through several rounds of column chromatography and/or high-performance liquid chromatography (HPLC). The column chromatography was conducted with 70–320 μm of silica gel 60 (Merck Kieselgel 60, Darmstadt, Germany), 230–400 μm of silica gel 60 (Merck Kieselgel 60, Darmstadt, Germany), 40–63 μm of C-18 (Merck Kieselgel 60 RP-18, Darmstadt, Germany), or 25–100 μm of Sephadex LH-20 (GE Healthcare, Göteborg, Sweden). The semi-preparative HPLC was performed using a Shimadzu LC-20 AD system (Shimadzu, Kyoto, Japan) equipped with an autosampler (SIL-20 AC), a column oven (CTO-20A), a photodiode array detector (SPD-M20A), and liquid chromatography with a COSMOSIL C_18_ column (4.6 × 150 mm, 5 μm) (Nacalai Tesque Inc., Kyoto, Japan). For chemical structure elucidation, mass spectra were recorded on an electrospray ionization-quadrupole-quadrupole-time-of-flight-mass spectrometer (Bruker ESI-QqTOF-MS, Manchester, UK). ^1^H nuclear magnetic resonance (NMR) (600 MHz) and ^13^C NMR (125 MHz) spectra were recorded on an NMR spectrometer (Bruker Avance III^TM^ HD 600 MHz, Graduate School of Pharmaceutical Sciences, Kyushu University).

The methanolic extract was fractioned through a Sephadex LH-20 column and eluted with acetone to give six fractions (A–F). Fraction A (2.57 g) was separated by reverse-phase column chromatography (C-18, gradient mixture of MeOH-H_2_O) to yield six fractions (A1–A6). Fraction A3 (24.5 mg) was fractioned on the reverse-phase column chromatography to yield four fractions (A3.1–A3.4). Compound **1** (2.7 mg) was obtained from fraction A3.2. Fraction B (6.85 g) was fractioned by a Sephadex LH-20 column and eluted with MeOH to give eight fractions (B1-B8). Under the same fractionation system, fraction B1 (2.04 g) was divided into three fractions, in which fraction B1.2 (1.81 g) was further purified on a semi-preparative HPLC column chromatography (C-18, gradient mixture of MeOH-H_2_O) to gain compound **2** (6.7 mg). Fractions B3 (627.1 mg), B4 (817.5 mg), B5 (500.9 mg), and B6 (126.7 mg) were individually separated by a Sephadex LH-20 column eluted with MeOH and further purified on a column chromatography (silica gel, gradient mixture of hexane-acetone) to obtain compounds **3** (144.0 mg), **4** (6.7 mg), **5** (9.2 mg), and **6** (3.0 mg), respectively.

### 2.3. Cell Culture and Adipocyte Differentiation

The mouse embryonic 3T3-L1 pre-adipocytes purchased from the American Type Culture Collection (ATCC, Manassas, VA, USA) were cultured at 37 °C with a 5% CO_2_ stream in a complete Dulbecco’s modified Eagle medium (DMEM) (Gibco, Gaithersburg, MA, USA) that contained 10% fetal bovine serum (FBS; Gibco, Gaithersburg, MA, USA), 1000 units mL^−1^ of penicillin/streptomycin (Gibco, Gaithersburg, MA, USA), and 2 mM of l-glutamine (Gibco, Gaithersburg, MA, USA). For adipocyte differentiation, 3T3-L1 cells at 100% confluent were incubated at 37 °C with a 5% CO_2_ stream for two days in a differentiation medium made of the complete DMEM plus 0.5 mM isobutyl methylxanthine (IBMX) (Sigma Aldrich, St. Louis, MO, USA), 1 µM of dexamethasone (Sigma Aldrich, St. Louis, MO, USA), and 5 µg mL^−1^ of insulin (Sigma Aldrich, St. Louis, MO, USA). Then, the medium was replaced with an insulin medium made of the complete DMEM plus 5 µg mL^−1^ of insulin, and cells were continuously incubated under the same conditions for another two days. The medium was replaced with the complete DMEM every two days to maintain adipocyte development. Cellular lipid droplets developed along the differentiation program could be observed under a light microscope (Nikon Ts2, Tokyo, Japan).

### 2.4. Cytotoxicity Assessments

The cytotoxic effect of elucidated compounds from *D*. *heterocarpum* on 3T3-L1 cell viability was evaluated using 3-(4,5-dimethylthiazol-2-yl)-2,5-diphenyl tetrazolium bromide (MTT) (Sigma Aldrich, St. Louis, MO, USA) assay. 3T3-L1 cells were seeded in 96-well plates at a density of 2 × 10^3^ cells/well and incubated at 37 °C with a 5% CO_2_ stream until reaching 100% confluent. Cells were then exposed to varying concentrations (5, 10, and 20 µM) of the tested compounds in the complete DMEM for 48 h. Before testing, every tested compound resuspended in absolute dimethyl sulfoxide (DMSO) (Sigma Aldrich, St. Louis, MO, USA) was prepared by dilution using the complete DMEM. The concentration of DMSO in each treatment was limited to <0.5% (*v*/*v*). DMSO at 0.5% (*v*/*v*) and 20 µM of oxyresveratrol (a known compound with the ability to suppress adipocyte differentiation [29]) were included in the tests as untreated vehicle and positive controls, respectively. After exposure for 48 h, the medium was removed, and the MTT solution (0.45 mg mL^−1^), prepared by dissolving in phosphate-buffered saline (PBS), was added. The assay was incubated at 37 °C with a 5% CO_2_ stream for 3 h. Then, the MTT solution was replaced with absolute DMSO to dissolve the purple formazan crystal that formed, which was further measured for absorbance at a 570 nm wavelength using a microplate reader (Anthros, Durham, NC, USA). The relative absorbance value between treated cells to vehicle control cells was presented as the percentage of cell viability. Non-cytotoxic concentrations of a tested compound were defined based on no significant difference in the percentages of cell viability derived from the treatments and the vehicle control.

3T3-L1 cell death after exposure to varying concentrations of the tested compounds was also determined by the nuclear staining technique. Hoechst 33342 (Sigma Aldrich, St. Louis, MO, USA) and propidium iodide (PI) (Sigma Aldrich, St. Louis, MO, USA) were used as staining dyes. After 48 h of treatment, the assay medium was removed from 3T3-L1 cells and replaced with PBS containing 2 µg mL^−1^ of Hoechst 33342 and 1 µg mL^−1^ of PI. Cells were stained for 30 min at 37 °C with a 5% CO_2_ stream and observed/photographed under a fluorescence microscope (Olympus IX51 with DP70, Tokyo, Japan). Bright cells with a disseminated nucleus under both blue and red filters were referred to as dead cells.

### 2.5. Quantification of Cellular Lipid Content

Oil Red O staining was performed to determine the content of lipid droplets in differentiating 3T3-L1 cells. The adipocyte differentiation program as described previously was established, and cells were exposed to each tested compound at 5 µM for 48 h after obtaining 100% confluence. Cells exposed to 0.5% (*v*/*v*) DMSO and 20 µM of oxyresveratrol served as untreated vehicle and positive controls, respectively. Tested compounds were diluted in the differentiation medium before testing, in which the concentrations of DMSO were restricted to be lower than 0.5% (*v*/*v*). After the treatments, differentiating 3T3-L1 cells on day 8 of the differentiation program were fixed with 10% formalin and incubated with the Oil Red O staining solution (Sigma Aldrich, St. Louis, MO, USA) at an ambient temperature for 1 h. Cells were then washed thrice with distilled water and 60% (*v*/*v*) isopropanol (Sigma Aldrich, St. Louis, MO, USA) to remove the excess dye. Oil Red O-stained 3T3-L1 cells were observed using a light microscope (Nikon Ts2, Tokyo, Japan). The cellular Oil Red O content was extracted using absolute isopropanol and measured for absorbance at a 510 nm wavelength by the microplate reader. The percentage of Oil Red O-stained cells was calculated after normalization with the total cellular protein content determined by a BCA protein assay kit (Thermo Scientific, Rockford, CA, USA).

### 2.6. Western Blot Analysis

The impacts of a tested compound on the expression and activation of proteins that are linked to cellular responses in the early phase of adipocyte differentiation were assessed using Western blot analysis. The list of proteins analyzed in this study included adipogenic regulators (i.e., PPARγ and C/EBPα) and the MAPK signaling pathways-mediated proteins (i.e., JNK, ERK, and p38). The expression or activation of each target protein was normalized with β-Actin as a reference cellular protein. The activation of JNK, ERK, and p38 was assessed using the phosphorylation ratio between those phosphorylated (i.e., p-ERK, p-JNK, and p-p38) and the sum of those phosphorylated and non-phosphorylated.

3T3-L1 pre-adipocytes at 100% confluence were exposed for 48 h to a tested compound at non-cytotoxic concentrations prepared in the differentiation medium. Cells were then washed with PBS (pH 7.4) and used for protein extraction by incubation in an ice-cold radio-immunoprecipitation assay buffer (Thermo Scientific, Rockford, CA, USA) supplemented with the protease inhibitor cocktail (Roche Applied Science, Indianapolis, IN, USA) for 45 min. Derived cell lysates were centrifuged at 4 °C, 6440× *g* for 15 min, and the supernatant was collected. The BCA protein assay kit was conducted to quantify the total protein content in the supernatant.

The same amount (30 µg) of protein from each treatment was loaded and separated onto 10% (*w*/*v*) sodium dodecyl sulfate-polyacrylamide (Bio-Lad Laboratories, Hercules, CA, USA) gel electrophoresis. The separated proteins were then transferred onto nitrocellulose membranes (Bio-Rad Laboratories, Hercules, CA, USA), which were blocked with 5% skim milk (Sigma Aldrich, St. Louis, MO, USA) in tris-buffered saline with 0.1% tween 20 (TBST) (pH 7.2). The membranes were then immunoblotted with specific primary antibodies, including PPARγ (1:1000), C/EBPα (1:1000), ERK (1:1000), p-ERK (1:1000), JNK (1:1000), p-JNK (1:1000), p38 (1:1000), p-p38 (1:1000), and β-Actin (1:1000) (Cell Signaling Technology, Danvers, MA, USA) at 4 °C for overnight. After washing thrice (~7 min) with TBST, the membranes were incubated for 2 h with the specific horseradish peroxidase-linked secondary antibody. The target protein bands and their intensities on the membranes were visualized, captured, and quantified under UV light after adding chemiluminescent substrates (Bio-Rad Laboratories, Hercules, CA, USA), using Chemiluminescent ImageQuant LAS 4000 (GE Healthcare Bio-Sciences AB, Uppsala, Sweden).

### 2.7. Statistical Analysis

All data were obtained from three independent experiments and reported as means ± standard deviations. Statistical analysis was performed with a one-way analysis of variant (ANOVA) using GraphPad Prism 9.3.1 (GraphPad Software Inc., San Diego, CA, USA). Differences in means at *p <* 0.05 were considered statically significant.

## 3. Results

### 3.1. Secondary Metabolites in the Methanolic Extracts of D. heterocarpum

**Figure 1:**
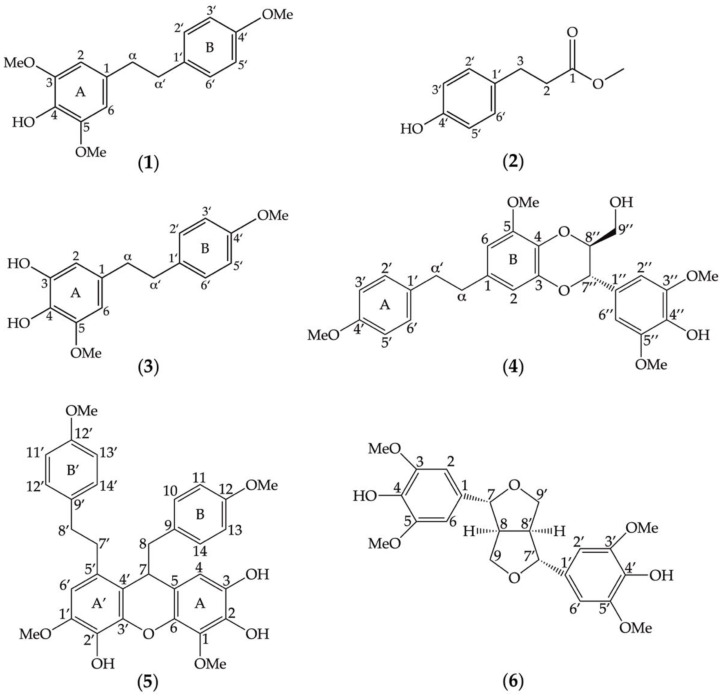
Chemical structures of secondary metabolites eluted from the methanolic extracts of *Dendrobium heterocarpum*. Six compounds (**1**–**6**) were assigned, and their structures were determined based on spectroscopic data in comparison with previously reported structures such as amoenylin (**1**), methyl 3-(4-hydroxyphenyl) propionate (**2**), 3,4-dihydroxy-5,4´-dimethoxybibenzyl (**3**), dendrocandin B (**4**), dendrofalconerol A (**5**), and syringaresinol (**6**).

The chemical structures of the six compounds (**1**–**6**) extracted from *D*. *heterocarpum* (Figure 1) were elucidated using their spectroscopic data in comparison with previously reported structures [30,31,32,33,34,35]. The description of each compound was listed below.

Compound **1** (Figure 1) was identified as amoenylin [30]: Yellow–brown amorphous solid; HR-ESIMS: *m/z* 311.1260 [M + Na]^+^ calculated for 311.1259 suggesting C_17_H_20_O_4_Na. ^1^H-NMR (300 MHz, acetone-*d*_6_) δ: 5.36 (H-4, s), 3.79 (3H, s, OMe-4′), 3.84 (6H, s, OMe-3, OMe-5), 6.35 (2H, s, H-2, H-6), 6.82 (2H, d, *J* = 9.0 Hz, H-3′, H-5′), 7.07 (2H, d, *J* = 8.4 Hz, H-2′, H-6′), 2.83 (4H, m, H-α, H-α′); ^13^C-NMR (75 MHz, acetone-*d*_6_) δ: 37.2 (C-α′), 38.3 (C-α), 55.2 (OMe-4′), 56.2 (OMe-3, OMe-5), 105.0 (C-1, C-6), 113.6 (C-3′, C-5′), 129.3 (C-2′, C-6′), 132.4 (C-4), 132.6 (C-1′), 133.4 (C-1), 147.0 (C-3, C-5), 157.9 (C-4′).

Compound **2** (Figure 1) was identified as methyl 3-(4-hydroxyphenyl) propionate [31]: White powder; HR-ESIMS: *m/z* 203.0665 [M + Na]^+^ calculated for 203.0684 suggesting C_10_H_12_O_3_Na. ^1^H-NMR (300 MHz, acetone-*d*_6_) δ: 2.53 (2H, t, *J* = 7.8 Hz, H-2), 2.79 (2H, t, *J* = 7.8 Hz, H-3), 3.58 (3H, s, OMe-1), 6.73 (2H, d, *J* = 8.4 Hz, H-3′, H-5′), 7.03 (2H, d, *J* = 8.4 Hz, H-2′, H-6′); ^13^C-NMR (75 MHz, acetone-*d*_6_) δ: 29.8 (C-3), 35.6 (C-2), 52.3 (OMe-1), 115.1 (C-3′, C-5′), 129.1 (C-2′, C-6′), 131.4 (C-1′), 155.7 (C-4′) 172.6 (C-1).

Compound **3** (Figure 1) was identified as 3,4-dihydroxy-5,4′-dimethoxybibenzyl [32]: Brown amorphous solid; HR-ESIMS: *m/z* 297.1127 [M + Na]^+^ calculated for 297.1102 suggesting C_16_H_18_O_4_Na. ^1^H-NMR (300 MHz, acetone-*d*_6_) δ: 2.53 (2H, t, *J* = 7.8 Hz, H-2), 2.79 (2H, t, *J* = 7.8 Hz, H-3), 3.58 (3H, s, OMe-1), 6.73 (2H, d, *J* = 8.4 Hz, H-3′, H-5′), 7.03 (2H, d, *J* = 8.4 Hz, H-2′, H-6′); ^13^ C-NMR (75 MHz, acetone-*d*_6_) δ: 29.8 (C-3), 35.6 (C-2), 52.3 (OMe-1), 115.1 (C-3′, C-5′), 129.1 (C-2′, C-6′), 131.4 (C-1′), 155.7 (C-4′), 172.6 (C-1).

Compound **4** (Figure 1) was identified as dendrocandin B [33]: White powder; HR-ESIMS: *m/z* 505.1850 [M + Na]^+^ calculated for 505.1838 suggesting C_27_H_30_O_8_Na. ^1^H-NMR (300 MHz, acetone-*d*_6_) δ: 2.84 (4H, m, H-α, H-α′), 3.54 (2H, m, H-9″), 3.80 (3H, m, OMe-4′), 3.83 (2H, m, H-9″), 3.87 (3H, s, OMe-5), 3.99 (6H, s, OMe-3″, OMe-5″), 4.02 (2H, m, H-8″), 4.96 (2H, d, *J* = 8.1 Hz, H-7″), 6.33 (2H, d, *J* = 1.8 Hz, H-6), 6.53 (2H, d, *J* = 1.8 Hz, H-2), 6.69 (2H, s, H-2″, H-6″), 7.11 (4H, d, H-2′, H-6′); ^13^C-NMR (75 MHz, acetone-*d*_6_) δ: 37.2 (C-α′), 38. 2 (C-α), 55.5 (OMe-4′), 56.3 (OMe-5), 56.6 (OMe-3″, OMe-5″), 105.0 (C-1, C-6), 113.6 (C-3′, C-5′), 129.3 (C-2′, C-6′), 132.4 (C-4), 61.8 (C-9″), 76.7 (C-7″), 78.5 (C-8″), 104.4 (C-6), 105.1 (C-2″,C-6″), 109.8 (C-2), 127.6 (C-1″), 129.6 (C-2′,C-6′), 131.2 (C-4), 134.0 (C-1′), 144.4 (C-3), 147.5 (C-3″, C-5″), 158.1 (C-4′).

Compound **5** (Figure 1) was identified as dendrofalconerol A [34]: Brown amorphous solid; HR-ESIMS: *m/z* 567.2003, [M + Na]^+^ calculated for C_32_H_32_O_8_Na; 567.1994, suggesting C_32_H_32_O_8_. ^1^H NMR (300 MHz, acetone-*d*_6_) δ: 2.65-2.71 (2H, m, H8), 2.74-2.83 (2H, m, H-8), 2.75-2.84 (2H, m, H-8′), 2.78-2.86 (2H, m, H-7′), 2.80-2.86 (2H, m, H-8′), 2.87-2.90 (2H, m, H-7′), 3.70 (3H, s, MeO-12), 3.73 (3H, s, MeO-12′), 3.81 (3H, s, MeO-1′), 3.89 (3H, s, MeO-1), 4.09 (1H, dd, *J* = 5.7, 6.9 Hz, H-7), 6.13 (1H, s, H-4), 6.60 (2H, d, *J* = 8.4 Hz, H-10, H-14), 6.65 (2H, s, H-6′), 6.68 (2H, d, *J* = 8.4 Hz, H-11, H-13), 6.82 (2H, d, *J* = 8.4 Hz, H-11′, H-13′), 7.13 (2H, d, *J* = 8.5 Hz, H-10′, H-14′); ^13^C NMR (75 MHz, acetone-*d*_6_) δ: 34.4 (C-7′), 37.5 (C-8′), 39.6 (C-7), 45.3 (C-8), 55.3 (MeO-12), 55.4 (MeO-12′), 56.2 (MeO-1′), 61.1 (MeO-1), 109.7 (C-4), 108.4 (C-6′), 113.9 (C-11, C-13), 117.8 (C- 5), 119.0 (C-4′), 129.5 (C-5′), 130.1 (C-10′, C-14′), 131.3 (C-10, C-14), 131.5 (C-9), 134.0 (C-2′), 134.6 (C-9′), 136.4 (C- 1), 137.3 (C-2), 139.9 (C-6), 142.3 (C-3′), 147.1 (C-1′), 158.9 (C-12′), 159.1 (C-12).

Compound **6** (Figure 1) was identified as syringaresinol [35]: Yellow–brown amorphous solid; HR-ESIMS: *m/z* 441.1527, [M + Na]^+^ calculated for C_22_H_26_O_8_Na; 441.1527, suggesting C_22_H_26_O_8_. ^1^H NMR (300 MHz, acetone-*d*_6_) δ: 3.07 (2H, m, H-8, H-8′), 3.81 (12H, s, MeO-3, MeO-5, MeO-3′, MeO-5′), 4.21 (4H, m, H-9, H-9′), 4.65 (4H, m, H-7, H-7′), 6.67 (8H, s, H-2, H-2′, H-6, H-6′); ^13^C NMR (75 MHz, acetone-*d*_6_) δ: 54.3 (C-6, C-6′), 54.3 (C-8, C-8′), 55.7 (MeO-3, MeO-5), 55.7 (MeO-3′, MeO-5′), 71.4 (C-9, C-9′), 85.8 (C-7, C-7′), 103.5 (C-2, C-2′), 132.2 (C-1, C-1′), 147.7 (C-3, C-3′), 147.7 (C-5, C-5′), 157.8 (C-5, C-5′).

### 3.2. Roles of Identified Phytochemicals in the Growth and Development of Adipocytic Cells

**Figure 2:**
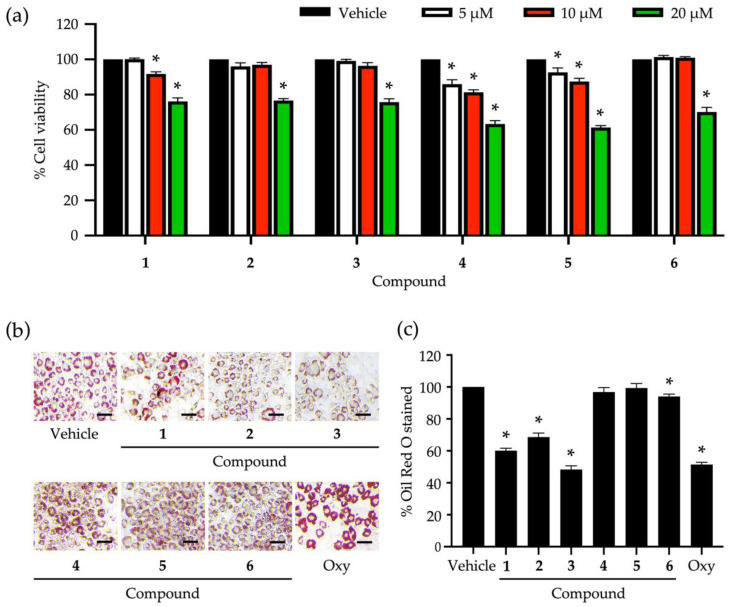
Roles of secondary metabolites eluted from *Dendrobium heterocarpum* in the viability and lipid storage of adipocytic cells. The percentage of cell viability was accounted for after treating 3T3-L1 cells with varying tested concentrations (5, 10, and 20 µM) of compounds **1**–**6** for 48 h (**a**). The impact of compounds **1**–**6** at the concentration of 5 µM on the lipid storage of 3T3-L1 cells was assessed using the Oil Red O staining method (**b**); scale bars = 20 µm. The percentage of Oil Red O stained was computed after the normalization with the total cellular protein (**c**). The graphical results demonstrate means ± SDs derived from three independent experiments. The asterisk (*) represents a statistical difference at *p* < 0.05 between the treatment and untreated vehicle control made of 0.5% (*v*/*v*) dimethyl sulfoxide, assessed by one-way ANOVA with Tukey’s post hoc test. A positive control made of 20 µM of oxyresveratrol (Oxy) might be included in some experiments.

Six identified compounds (**1**–**6**) were tested for their cytotoxic effects on 3T3-L1 pre-adipocytes (Figure 2a). The lowest percentage of cell viability (61.32 ± 1.1%) was observed after treating 3T3-L1 cells with compounds **4** and **5** at 20 µM. Even the lowest tested concentration (5 µM) of these compounds still posed cytotoxic effects on 3T3-L1 cells, supported by the significant decrease in cell viability compared to untreated vehicle controls (Figure 2a). Compounds **2**, **3**, and **6** demonstrated similar cytotoxicity, giving the same range of non-cytotoxic concentrations at ≲10 µM. The biological impacts of the six compounds at the same concentration (5 µM) on the cellular lipid storage in 3T3-L1 cells were also assessed with the Oil Red O staining technique (Figure 2b,c). In comparison with the untreated vehicle control, the decrease in the cellular lipid storage of 3T3-L1 cells was apparently observed after treating the cells with compounds **1**, **2**, and **3** (Figure 2b). A statistical comparison proved that compounds **1**, **2**, **3**, and **6** could significantly decrease the percentage of Oil Red O-stained 3T3-L1 cells (Figure 2c), while compound **3** posed the maximum reduction (51.73 ± 2.31%) compared to the others and controls. Besides, compound **3** at 5 µM could suppress the cellular lipid accumulation at the same level as that observed with a positive control made of 20 µM of oxyresveratrol (Figure 2c).

**Figure 3:**
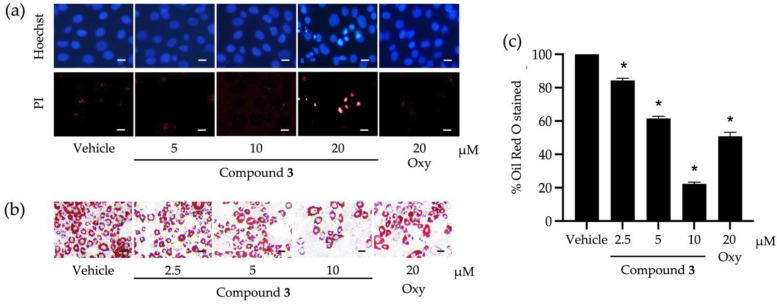
Roles of compound **3** derived from *Dendrobium heterocarpum* in the viability and lipid storage of adipocytic cells. Dead 3T3-L1 cells caused by compound **3** tested at different concentrations (5, 10, and 20 µM) were confirmed by a nuclear staining method using Hoechst 33342 (Hoechst) and propidium iodide (PI) (**a**). The impact of compound **3** at non-cytotoxic concentrations (2.5, 5, and 10 µM) on the lipid storage of 3T3-L1 cells was assessed using the Oil Red O staining method (**b**); scale bars = 10 µm. The percentage of Oil Red O-stained was computed after the normalization with the total cellular protein (**c**). The graphical results demonstrate means ± SDs derived from three independent experiments. The asterisk (*) represents a statistical difference at *p* < 0.05 between the treatment and untreated vehicle control made of 0.5% (*v*/*v*) dimethyl sulfoxide, assessed by one-way ANOVA with Tukey’s post hoc test. A positive control made of 20 µM of oxyresveratrol (Oxy) might be included in some experiments.

Compound **3,** with the greatest inhibitory activity against the lipid storage in 3T3-L1 cells, was investigated further for its possible molecular mechanisms that regulate adipocyte differentiation. The cytotoxic effect of compound **3** at different tested concentrations (Figure 2a) was confirmed with a nuclear straining approach (Figure 3a). It was explicit that some dead cells stained with Hoechst 33342 were observed after testing with compound **3** at 20 µM (Figure 3a). Non-cytotoxic concentrations (2.5, 5, and 10 µM) of this compound were tested for the capability to suppress cellular lipid storage in 3T3-L1 cells using the Oil Red O staining method (Figure 3b,c). The cellular lipid content was significantly decreased when the tested concentration of compound **3** increased (Figure 3c). Based on the percentage of Oil Red O-stained (Figure 3c), the half-maximal inhibitory concentration (IC_50_) to suppress cellular lipid accumulation of compound **3** was computed and determined at 6.30 ± 0.10 µM. This IC_50_ value was approximately 3-fold lower than that of the positive control made of oxyresveratrol (20.20 ± 1.20 µM).

**Figure 4:**
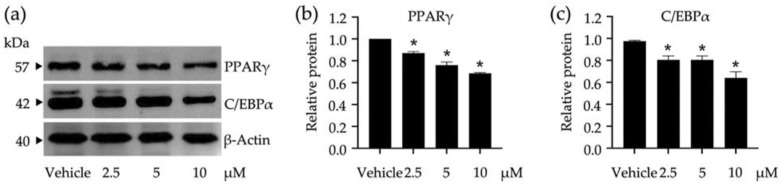
Impacts of compound **3** on the peroxisome proliferators activated receptor γ (PPARγ) and CCAAT/enhancer-binding protein α (C/EBPα) expression in 3T3-L1 cells. The band intensities of these transcription factors were visualized by Western blotting assay (**a**) and used for calculating their expression relative to β-Actin as a reference protein (**b**,**c**). Non-cytotoxic concentrations (2.5, 5, and 10 µM) of compound **3** were used in this study. The graphical results demonstrate means ± SDs derived from three independent experiments. The asterisk (*) represents a statistical difference at *p* < 0.05 between the treatment and untreated vehicle control made of 0.5% (*v*/*v*) dimethyl sulfoxide, assessed by one-way ANOVA with Tukey’s post hoc test.

To elucidate certain underlying molecular mechanisms of compound **3** in adipocyte differentiation, the expression levels of adipogenic regulators (i.e., PPARγ and C/EBPα) were tracked at early adipocyte differentiation using the Western blot analysis (Figure 4). The band intensities of both transcription factors were gradually decreased when the tested concentrations of compound **3** increased (Figure 4a). This observation was consistent with the expression levels of these transcription factors relative to β-Actin as a reference protein (Figure 4b,c). Compound **3** at 10 µM offered the maximum suppression of both transcription factors compared to the treatments at lower concentrations and the untreated vehicle control. The decreased expression levels of PPARγ and C/EBPα also correlated with the decrease in lipid accumulation of 3T3-L1 cells treated with this compound (Figure 3c).

**Figure 5:**
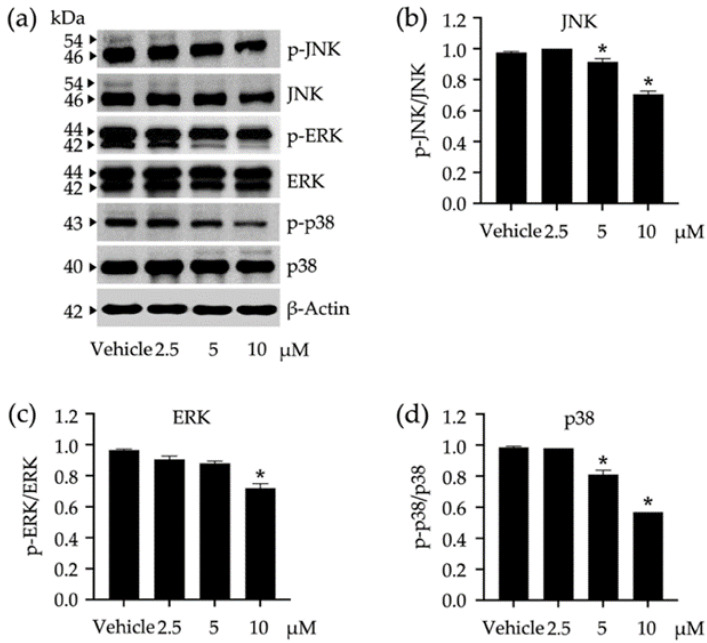
Impacts of compound **3** on the activation of c-Jun N-terminal kinase (JNK), extracellular signal-regulated kinase (ERK), and stress-activated protein kinase (p38) in 3T3-L1 cells. The band intensities of these kinases were visualized by Western blotting assay (**a**) and used for calculating their expression relative to β-Actin as a reference protein. The expression ratio between phosphorylated analog (p-JNK, p-ERK, and p-p38) and the summary (phosphorylated + non-phosphorylated) of each kinase was accounted for to describe the activation level (**b**–**d**). Non-cytotoxic concentrations (2.5, 5, and 10 µM) of compound **3** were used in this study. The graphical results demonstrate means ± SDs derived from three independent experiments. The asterisk (*) represents a statistical difference at *p* < 0.05 between the treatment and untreated vehicle control made of 0.5% (*v*/*v*) dimethyl sulfoxide, assessed by one-way ANOVA with Tukey’s post hoc test.

As the MAPK signaling pathways exert their significant roles in regulating several cellular responses such as proliferation, differentiation, survival, and apoptosis, the impact of a tested compound on the modulation of these pathways may offer a promising way to monitor adipocyte differentiation. Compound **3** was tested for its role in the activation of JNK/ERK/p38-mediated MAPK signaling pathways (Figure 5). The activation of these kinases was tracked through phosphorylation using Western blot analysis (Figure 5a), in which the phosphorylated ratio for each protein was accounted for after treating 3T3-L1 cells with varying concentrations of compound **3** (Figure 5b–d). In comparison to untreated vehicle controls, compound **3** at 10 µM significantly suppressed the phosphorylation of every tested protein, while this compound at 2.5 µM did not affect the phosphorylation of any tested proteins. JNK and p38 phosphorylation were more susceptible to compound **3** than that observed with ERK (Figure 5b–d).

## 4. Discussion

The genus *Dendrobium* has been reported as a rich source of valuable metabolites with potent bioactivity to suppress adipocyte differentiation—a critical biological process to indicate obesity [20,26,27,36]. Our study also proved that *D*. *heterocarpum*, a member of the genus *Dendrobium* and a widespread Thai orchid species, houses a variety of phytochemicals with inhibitory activity against adipocyte development. Six compounds (**1**–**6**) were profiled in *D*. *heterocarpum* methanolic extracts. An orchid bibenzyl, amoenylin (compound **1**), was firstly elucidated from the methanolic extract of *Dendrobium amoenum* [30]. This compound seemed to be a common metabolite found in the genus *Dendrobium*, while its bioactivity to suppress cellular lipid accumulation was firstly unveiled here. Methyl 3-(4-hydroxyphenyl) propionate (compound **2**) is a prevalent plant secondary metabolite previously reported to be found in the ethyl acetate extract of *Amyris texana* leaves and the methanolic extract of an orchid species, *Bulbophyllum retusiusculum* [31]. In addition to its recognized plant growth-related function as a biological nitrification inhibitor [37], this phenylpropanoid was firstly reported here with less of a cytotoxic effect on 3T3-L1 pre-adipocytes (compared to compounds **1**, **4**, and **5** eluted in this work) and the capability to reduce lipid storage of these cells.

Compound **3**, 3,4-dihydroxy-5,4′-dimethoxybibenzyl, is another bibenzyl derived from *D*. *heterocarpum*, which showed the greatest bioactivity to inhibit lipid accumulation in 3T3-L1 cells. This orchid bibenzyl was firstly elucidated from *Dendrobium moniliforme* with a given name, moniliformine (unapproved name) [32], which was also isolated later from *Dendrobium harveyanum* [20] and *Dendrobium officinale* [38]. This compound posed some potent bioactivities to impede the invasive mechanisms of non-small-cell lung cancer cells and sensitize the apoptosis of these cancer cells [39,40], but did not show a cytotoxic effect on the HeLa cervical cancer cell line [38]. In 2008, the sister molecule of compound **3**, dendrocandin A, was isolated from *Dendrobium candidum* [33]. The authors also found dendrocandin B (compound **4**) from this *Dendrobium* species [33], which was also elucidated in this study. Compound **4** was formerly reported to have inhibitory activity against diverse human cancer cells [38,41], but did not hamper α-glucosidase and pancreatic lipase activities [20]. Lacking such an enzyme-inhibiting function of compound **4** might be linked to the absence of its bioactivity to suppress lipid accumulation in 3T3-L1 cells tested in this study.

Dendrofalconerol A (compound **5**) was firstly isolated from *Dendrobium falconeri* [34] and further found in diverse *Dendrobium* species [17,20,41]. This bisbibenzyl was recognized for its anticancer potential [42,43] and its strong capability to inhibit α-glucosidase and pancreatic lipase activities [17,20]. However, based on our findings, compound **5** did not exhibit the inhibitory effect on lipid accumulation in 3T3-L1 cells, which was contrary to its sister molecule, dendrofalconerol B [20]. These results prove that the varied biological functions of chemicals rely significantly on their molecular structures with different radical groups and isomeric analogs. This molecular structure-dependent bioactivity was also observed with compounds **1** and **3**. These two compounds are bibenzyls that have only one different radical group by replacing a methoxy group at the C-3 on the A ring in compound **1** with a hydroxyl group in compound **3**. The replacement led to the decreased cytotoxic effect on 3T3-L1 cells (Figure 2a) and the increased capability to inhibit lipid accumulation in these cells (Figure 2c). This methoxy/hydroxyl replacement in a parent compound has been proposed to link with the shift in various bioactivities, including lipogenesis and lipolysis during adipocyte differentiation [44,45,46,47]. Compound **6**, syringaresinol, was isolated from *Tripterygium wilfordii*, and it was structurally elucidated for the first time in 1976 [48]. Syringaresinol is a member of lignans, which has been increasingly reported to be found in several *Dendrobium* species [35,49,50,51]. This lignan has been proven recently to have no function to promote glucose uptake in 3T3L-1 cells [52]. We found here that syringaresinol slightly affected lipid storage in 3T3-L1 cells compared to compounds **1**–**3** (Figure 2c), and even the influencing level was significantly different from that observed with the untreated vehicle control.

Adipose tissues are essential parts of the body and have some critical functions to maintain body temperature, storage energy, and protect viscera. The formation of adipose tissue requires regular growth and the development of adipocytes. Adipocyte differentiation is a key step to convert pre-adipocytes to mature adipocytes that fully function in cellular lipid metabolism and adipose tissue development [53,54]. Excess adipocyte differentiation can cause irregularities in adipose tissue formation and body energy homeostasis, leading to the incidence of obesity and other metabolism-related diseases [55]. Hence, adipocyte differentiation is a promising platform to understand the emergence of obesity and search for a pharmacotherapeutic way to prevent or monitor this disease. There are many molecular mechanisms taking place during adipocyte differentiation and regulating or maintaining this biological process [12]. Compound **3** already proved that it could strongly suppress the lipid accumulation in 3T3-L1 cells. One of the underlying molecular mechanisms is that compound **3** could downregulate a set of crucial transcription factors (i.e., PPARγ and C/EBPα) triggered during the early differentiation of 3T3-L1 cells. These transcription factors play a significant role in adipocyte differentiation by regulating lipogenesis and cellular lipid storage. A study reported that *Pparg*-knocked out mice had impaired lipid metabolisms and were resistant to high-fat diet-induced obesity [56]. Suppressed adipocyte differentiation and impaired lipid metabolisms were unveiled from *C/ebpa*-deleted 3T3-L1 cells and mice [57].

It was also proved here that compound **3** could suppress the activation of the MAPK signaling pathways (i.e., JNK, ERK, and p38) in 3T3-L1 cells. To date, the biological roles of these biomarkers in adipocyte differentiation are still rarely known and require further investigation. Some studies describe that JNK is implicated in the development of obesity-related insulin resistance, and ERK is required for cellular proliferation and differentiation [9,11,58]. The decrease in JNK and ERK phosphorylation observed in this study might not be a direct result of compound **3**. A study unveiled that the phosphorylation of JNK and ERK in 3T3-L1 cells was significantly activated after treating these cells with the chemokine (C-X-C motif) ligand 3 (CXCL3) [59]. The authors also found that the expression of C/EBPβ and δ (other adipogenic regulators) induced by CXCL3 was downregulated if JNK and ERK were inhibited by their specific inhibitors [59]. Hence, CXCL3 as a crucial adipokine in adipocyte differentiation might be negatively affected by compound **3**, which consequently caused the decrease in JNK and ERK phosphorylation. ERK has been reviewed, with a pivotal role in cell cycle progression and cell proliferation [9]. Many studies revealed that the decrease in ERK phosphorylation was responsible for the increase in apoptosis, autophagy, and metastasis of diverse cancer cells [60] and related references therein. Suppressing this transcription factor might restrain pre-adipocytes at the G0 phase of the mitotic clonal expansion, which is supposed to further limit hyperplasia in adipose tissue formation. The phosphorylation of p38—a stress-activated protein kinase—was also decreased in the presence of compound **3**. A study reported that high-fat diet-induced obesity was significantly diminished in mice administered the p38 inhibitor [61]. These p38 inhibitor-fed mice also had reduced macrophage infiltration into white adipose tissues and a lower level of tumor necrosis factor α, compared to untreated control mice. Thus, the decrease in p38 phosphorylation might offer a possible link to the modulation of adipocyte differentiation, which would further mitigate obesity.

Adipocyte differentiation involves the sequentially coordinated changes in hormonal sensitivity and a gene expression-accompanied morphological shift from fibroblasts to round-shaped cells. There are several genes and proteins triggered during adipocyte differentiation and maturation, some of which play a pivotal role in cellular lipid metabolism, known as adipogenic effectors (e.g., fatty acid synthase, perilipin 1, lipoprotein lipase, and adiponectin). Further investigations of how a natural molecule exerts its biological impact on the expression of these adipogenic effectors are also required to provide significant insights into its anti-adipogenic activity. The protein expression analysis in our study only describes the biological impact of a tested compound on the downstream central dogma of molecular biology. Additional studies at the transcription level (upstream) are also essential to elucidate the underlying molecular mechanisms of the tested molecule in-depth.

## 5. Conclusions

The *D*. *heterocarpum* methanolic extract houses diverse secondary metabolites. Among them, six compounds were identified, including amoenylin (**1**), methyl 3-(4-hydroxyphenyl) propionate (**2**), 3,4-dihydroxy-5,4′-dimethoxybibenzyl (**3**), dendrocandin B (**4**), dendrofalconerol A (**5**), and syringaresinol (**6**). These compounds had varying impacts on the viability and lipid accumulation of 3T3-L1 pre-adipocytes. Compound **3** exhibited the greatest reduction in 3T3-L1 lipid storage compared to the other compounds. This decreased cellular lipogenesis was proven to be linked to the downregulation of some key adipogenic regulators (i.e., PPARγ and C/EBPα) and the suppression of JNK/ERK/p38-mediated MAPK signaling pathways. To this end, *D*. *heterocarpum* proved its potential to be a natural source for discovering pharmaceutical products with promising bioactivities to modulate adipocyte differentiation. Compound **3** would be a potent candidate for further investigation and development as a phytomedicine for preventing and curing obesity.

## Data Availability

Data are contained within the article.

## References

[1] Ambele M.A., Dhanraj P., Giles R., Pepper M. (2020). Adipogenesis: A complex interplay of multiple molecular determinants and pathways. Int. J. Mol. Sci..

[2] De Lorenzo A., Romano L., Di Renzo L., Di Lorenzo N., Cenname G., Gualtieri P. (2020). Obesity: A preventable, treatable, but relapsing disease. Nutrition.

[3] Ma S., Xi B., Yang L., Sun J., Zhao M., Bovet P. (2021). Trends in the prevalence of overweight, obesity, and abdominal obesity among Chinese adults between 1993 and 2015. Int. J. Obes..

[4] Mark D.H. (2005). Deaths attributable to obesity. JAMA..

[5] Lee H.-W., Rhee D.-K., Kim B.-O., Pyo S. (2018). Inhibitory effect of sinigrin on adipocyte differentiation in 3T3-L1 cells: Involvement of AMPK and MAPK pathways. Biomed. Pharmacother..

[6] Jakab J., Miškić B., Miškić S., Juranić B., Ćosić V., Schwarz D., Včev A. (2021). Adipogenesis as a potential anti-obesity target: A review of pharmacological treatment and natural products. Diabetes Metab. Syndr. Obes..

[7] Cowherd R.M., Lyle R.E., McGehee R.E. (1999). Molecular regulation of adipocyte differentiation. Semin. Cell Dev. Biol..

[8] Rayalam S., Yang J.-E., Ambati S., Della-Fera M.A., Baile C.A. (2008). Resveratrol induces apoptosis and inhibits adipogenesis in 3T3-L1 adipocytes. Phytother. Res..

[9] Bost F., Aouadi M., Caron L., Binétruy B. (2005). The role of MAPKs in adipocyte differentiation and obesity. Biochimie.

[10] Prusty D., Park B.-H., Davis K.E., Farmer R.S. (2002). Activation of MEK/ERK signaling promotes adipogenesis by enhancing peroxisome proliferator-activated receptor γ (PPARγ) and C/EBPα gene expression during the differentiation of 3T3-L1 preadipocytes. J. Biol. Chem..

[11] Lim S.H., Lee H.S., Han H.-K., Choi C.-I. (2021). Saikosaponin A and D inhibit adipogenesis via the AMPK and MAPK signaling pathways in 3T3-L1 adipocytes. Int. J. Mol. Sci..

[12] Guru A., Issac P.K., Velayutham M., Saraswathi N.T., Arshad A., Arockiaraj J. (2021). Molecular mechanism of down-regulating adipogenic transcription factors in 3T3-L1 adipocyte cells by bioactive anti-adipogenic compounds. Mol. Biol. Rep..

[13] Lao W., Tan Y., Jin X., Xiao L., Kim J.J.Y., Qu X. (2015). Comparison of cytotoxicity and the anti-adipogenic effect of green tea polyphenols with epigallocatechin-3-gallate in 3T3-L1 preadipocytes. Am. J. Chin. Med..

[14] Lam Y., Ng T.B., Yao R.M., Shi J., Xu K., Cho Wing Sze S., Zhang K.Y. (2015). Evaluation of chemical constituents and important mechanism of pharmacological biology in *Dendrobium* plants. Evid.-Based Compl. Alt..

[15] Xiaohua J., Singchi C., Yibo L. (2009). Taxonomic revision of *Dendrobium moniliforme* complex (Orchidaceae). Sci. Hortic..

[16] Inthongkaew P., Chatsumpun N., Supasuteekul C., Kitisripanya T., Putalun W., Likhitwitayawuid K., Sritularak B. (2017). α-Glucosidase and pancreatic lipase inhibitory activities and glucose uptake stimulatory effect of phenolic compounds from *Dendrobium formosum*. Rev. Bras. Farmacogn..

[17] Limpanit R., Chuanasa T., Likhitwitayawuid K., Jongbunpresert V., Sritularak B. (2016). α-Glucosidase inhibitors from *Dendrobium tortile*. Rec. Nat. Prod..

[18] Liu Y., Yang L., Zhang Y., Liu X., Wu Z., Gilbert R.G., Deng B., Wang K. (2020). *Dendrobium officinale* polysaccharide ameliorates diabetic hepatic glucose metabolism via glucagon-mediated signaling pathways and modifying liver-glycogen structure. J. Ethnopharmacol..

[19] Lu Y., Kuang M., Hu G.-P., Wu R.-B., Wang J., Liu L., Lin Y.-C. (2014). Loddigesiinols G–J: α-Glucosidase inhibitors from *Dendrobium loddigesii*. Molecules.

[20] Maitreesophone P., Khine H.E.E., Quiel Lasam Nealiga J., Kongkatitham V., Panuthai P., Chaotham C., Likhitwitayawuid K., Sritularak B. (2022). α-Glucosidase and pancreatic lipase inhibitory effects and anti-adipogenic activity of dendrofalconerol B, a bisbibenzyl from *Dendrobium harveyanum*. S. Afr. J. Bot..

[21] Na Ranong S., Likhitwitayawuid K., Mekboonsonglarp W., Sritularak B. (2019). New dihydrophenanthrenes from *Dendrobium infundibulum*. Nat. Prod. Res..

[22] San H.T., Boonsnongcheep P., Putalun W., Mekboonsonglarp W., Sritularak B., Likhitwitayawuid K. (2020). α-Glucosidase inhibitory and glucose uptake stimulatory effects of phenolic compounds from *Dendrobium christyanum*. Nat. Prod. Commun..

[23] Sarakulwattana C., Mekboonsonglarp W., Likhitwitayawuid K., Rojsitthisak P., Sritularak B. (2020). New bisbibenzyl and phenanthrene derivatives from *Dendrobium scabrilingue* and their α-glucosidase inhibitory activity. Nat. Prod. Res..

[24] Sun J., Zhang F., Yang M., Zhang J., Chen J., Zhan R., Li L., Chen Y. (2014). Isolation of α-glucosidase inhibitors including a new flavonol glycoside from *Dendrobium devonianum*. Nat. Prod. Res..

[25] Thant M.T., Chatsupun N., Mekboonsonglarp W., Sritularak B., Likhitwitayawuid K. (2020). New fluorene derivatives from *Dendrobium gibsonii* and their α-glucosidase inhibitory activity. Molecules.

[26] Thant M.T., Khine H.E.E., Quiel Lasam Nealiga J., Chatsumpun N., Chaotham C., Sritularak B., Likhitwitayawuid K. (2022). α-Glucosidase inhibitory activity and anti-adipogenic effect of compounds from *Dendrobium delacourii*. Molecules.

[27] Li X.-W., Huang M., Lo K., Chen W.-L., He Y.-Y., Xu Y., Zheng H., Hu H., Wang J. (2019). Anti-diabetic effect of a shihunine-rich extract of *Dendrobium loddigesii* on 3T3-L1 cells and db/db mice by up-regulating AMPK–GLUT4–PPARα. Molecules.

[28] Vaddhanaphuti N.B. (2005). A Field Guide to the Wild Orchids of Thailand.

[29] Tan H.Y., Iris M.Y., Li E.T., Wang M. (2015). Inhibitory effects of oxyresveratrol and cyanomaclurin on adipogenesis of 3T3-L1 cells. J. Funct. Foods.

[30] Majumder P.L., Guha S., Sen P. (1999). Bibenzyl derivatives from the orchid *Dendrobium amoenum*. Phytochemistry.

[31] Fang Y.-S., Yang M.-H., Cai L., Wang J.-P., Yin T.-P., Yu J., Ding Z.-T. (2018). New phenylpropanoids from *Bulbophyllum retusiusculum*. Arch. Pharm. Res..

[32] Zhi-Ming B., Zheng-Tao W., Luo-Shan X. (2004). Chemical constituents of *Dendrobium moniliforme*. Acta Bot. Sin..

[33] Li Y., Wang C.-L., Guo S.-X., Yang J.-S., Xiao P.-G. (2008). Two new compounds from *Dendrobium candidum*. Chem. Pharm. Bull..

[34] Sritularak B., Likhitwitayawuid K. (2009). New bisbibenzyls from *Dendrobium falconeri*. Helv. Chim. Acta.

[35] Sritularak B., Duangrak N., Likhitwitayawuid K. (2011). A new bibenzyl from *Dendrobium secundum*. Z. Naturforsch. C.

[36] Qu J., Tan S., Xie X., Wu W., Zhu H., Li H., Liao X., Wang J., Zhou Z.-A., Huang S. (2021). *Dendrobium Officinale* polysaccharide attenuates insulin resistance and abnormal lipid metabolism in obese mice. Front. Pharmacol..

[37] Zakir H.A., Subbarao G.V., Pearse S.J., Gopalakrishnan S., Ito O., Ishikawa T., Kawano N., Nakahara K., Yoshihashi T., Ono H. (2008). Detection, isolation and characterization of a root-exuded compound, methyl 3-(4-hydroxyphenyl) propionate, responsible for biological nitrification inhibition by sorghum (*Sorghum bicolor*). New Phytol..

[38] Ren G., Deng W.Z., Xie Y.F., Wu C.H., Li W.Y., Xiao C.Y., Chen Y.L. (2020). Bibenzyl derivatives from leaves of *Dendrobium officinale*. Nat. Prod. Commun..

[39] Putri H.E., Nutho B., Rungrotmongkol T., Sritularak B., Vinayanuwattikun C., Chanvorachote P. (2021). Bibenzyl analogue DS-1 inhibits MDM2-mediated p53 degradation and sensitizes apoptosis in lung cancer cells. Phytomedicine.

[40] Putri H.E., Sritularak B., Chanvorachote P. (2021). DS-1 inhibits migration and invasion of non-small-cell lung cancer cells through suppression of epithelial to mesenchymal transition and integrin β1/FAK signaling. Anticancer Res..

[41] Mittraphab A., Muangnoi C., Likhitwitayawuid K., Rojsitthisak P., Sritularak B. (2016). A new bibenzyl-phenanthrene derivative from *Dendrobium signatum* and its cytotoxic activity. Nat. Prod. Commun..

[42] Pengpaeng P., Sritularak B., Chanvorachote P. (2015). Dendrofalconerol A sensitizes anoikis and inhibits migration in lung cancer cells. J. Nat. Med..

[43] Pengpaeng P., Sritularak B., Chanvorachote P. (2015). Dendrofalconerol A suppresses migrating cancer cells via EMT and integrin proteins. Anticancer Res..

[44] Kayser O., Kolodziej H. (1999). Antibacterial activity of simple coumarins: Structural requirements for biological activity. Z. Naturforsch. C.

[45] Zhang H., Yang F., Qi J., Song X.C., Hu Z.F., Zhu D.N., Yu B.Y. (2010). Homoisoflavonoids from the fibrous roots of *Polygonatum odoratum* with glucose uptake-stimulatory activity in 3T3-L1 adipocytes. J. Nat. Prod..

[46] Yang Y., Yang X., Xu B., Zeng G., Tan J., He X., Hu C., Zhou Y. (2014). Chemical constituents of *Morus alba* L. and their inhibitory effect on 3T3-L1 preadipocyte proliferation and differentiation. Fitoterapia.

[47] Nishina A., Ukiya M., Fukatsu M., Koketsu M., Ninomiya M., Sato D., Yamamoto J., Kobayashi-Hattori K., Okubo T., Tokuoka H. (2015). Effects of various 5, 7-dihydroxyflavone analogs on adipogenesis in 3T3-L1 cells. Biol. Pharm. Bull..

[48] Bryan R.F., Fallon L. (1976). Crystal structure of syringaresinol. J. Chem. Soc. Perkin Trans..

[49] Chen Y.G., Yu H., Lian X. (2015). Isolation of stilbenoids and lignans from *Dendrobium hongdie*. Trop. J. Pharm. Res..

[50] Hu J.M., Chen J.J., Yu H., Zhao Y.X., Zhou J. (2008). Two novel bibenzyls from *Dendrobium trigonopus*. J. Asian Nat. Prod. Res..

[51] Zhou J. (2015). Chemical constituents of *Dendrobium officinale*. Chin. Tradit. Herb. Drugs.

[52] Lv H.W., Li Y.X., Luo M., Qi J.M., Fu Z.F., Zhang H.J., Guo Y.Q., Chu C., Li H.B., Yan J.Z. (2021). Two new nor-lignans from *Selaginella pulvinata* (Hook. & Grev.) Maxim and their antihyperglycemic activities. Nat. Prod. Res..

[53] Poulos S.P., Dodson M.V., Hausman G.J. (2010). Cell line models for differentiation: Preadipocytes and adipocytes. Exp. Biol. Med..

[54] Morrison S., McGee S.L. (2015). 3T3-L1 adipocytes display phenotypic characteristics of multiple adipocyte lineages. Adipocyte.

[55] Li Y., Rong Y., Bao L., Nie B., Ren G., Zheng C., Amin R., Arnold R., Jeganathan R.B., Huggins K.W. (2017). Suppression of adipocyte differentiation and lipid accumulation by stearidonic acid (SDA) in 3T3-L1 cells. Lipids Health Dis..

[56] Jones J.R., Barrick C., Kim K.A., Lindner J., Blondeau B., Fujimoto Y., Shiota M., Kesterson R.A., Kahn B.B., Magnuson M.A. (2005). Deletion of PPARγ in adipose tissues of mice protects against high fat diet-induced obesity and insulin resistance. Proc. Natl. Acad. Sci. USA.

[57] Darlington G.J., Wang N., Hanson R.W. (1995). C/EBPα: A critical regulator of genes governing integrative metabolic processes. Curr. Opin. Genet. Dev..

[58] Tarantino G., Caputi A. (2011). JNKs, insulin resistance and inflammation: A possible link between NAFLD and coronary artery disease. World J. Gastroenterol..

[59] Kusuyama J., Komorizono A., Bandow K., Ohnishi T., Matsuguchi T. (2016). CXCL3 positively regulates adipogenic differentiation. J. Lipid Res..

[60] He L., Su Q., Bai L., Li M., Liu J., Liu X., Zhang C., Jiang Z., He J., Shi J. (2020). Recent research progress on natural small molecule bibenzyls and its derivatives in *Dendrobium* species. Eur. J. Med. Chem..

[61] Maekawa T., Jin W., Ishii S. (2010). The role of ATF-2 family transcription factors in adipocyte differentiation: Antiobesity effects of p38 inhibitors. Mol. Cell. Biol..

